# Understanding Cancer Risk Among Bangladeshi Women: An Explainable Machine Learning Approach to Socio-Reproductive Factors Using Tertiary Hospital Data

**DOI:** 10.3390/healthcare13121432

**Published:** 2025-06-15

**Authors:** Muhammad Rafiqul Islam, Humayera Islam, Syeda Masuma Siddiqua, Salman Bashar Al Ayub, Beauty Saha, Nargis Akter, Rashedul Islam, Nazrina Khatun, Andrew Craver, Habibul Ahsan

**Affiliations:** 1 Institute for Population and Precision Health, The University of Chicago, Chicago, IL 60637, USAhahsan@bsd.uchicago.edu (H.A.); 2 Department of Medical Oncology, National Institute of Cancer Research and Hospital, Dhaka 1212, Bangladesh; 3 Unity Through Population Service, Dhaka 1230, Bangladesh; 4 Department of Radiotherapy, Mymensingh Medical College & Hospital, Mymensingh 2200, Bangladesh; 5 Ahsania Mission Cancer and General Hospital, Dhaka 1230, Bangladesh; 6 Department of Public Health Sciences, Biological Science Division, The University of Chicago, Chicago, IL 60637, USA; 7 Department of Family Medicine, Biological Science Division, The University of Chicago, Chicago, IL 60637, USA

**Keywords:** breast cancer risk, explainable machine learning, women reproductive risk factors

## Abstract

Background: Breast cancer poses a significant health challenge in Bangladesh, where limited screening and unique reproductive patterns contribute to delayed diagnoses and subtype-specific disparities. While reproductive risk factors such as age at menarche, parity, and contraceptive use are well studied in high-income countries, their associations with hormone-receptor-positive (HR+) and triple-negative breast cancer (TNBC) remain underexplored in low-resource settings. Methods: A case-control study was conducted at the National Institute of Cancer Research and Hospital (NICRH) including 486 histopathologically confirmed breast cancer cases (246 HR+, 240 TNBC) and 443 cancer-free controls. Socio-demographic and reproductive data were collected through structured interviews. Machine learning models—including Logistic Regression, Lasso, Support Vector Machines, Random Forest, and XGBoost—were trained using stratified five-fold cross-validation. Model performance was evaluated using sensitivity, F1-score, and Area Under Receiver Operating Curve (AUROC). To interpret model predictions and quantify the contribution of individual features, we employed Shapley Additive exPlanation (SHAP) values. Results: XGBoost achieved the highest overall performance (F1-score = 0.750), and SHAP-based interpretability revealed key predictors for each subtype. Rural residence, low education (≤5 years), and undernutrition were significant predictors across subtypes. Cesarean delivery and multiple abortions were more predictive of TNBC, while urban residence, employment, and higher education were more predictive of HR+. Age at menarche and age at first childbirth showed decreasing predictive importance with increasing age for HR+, while larger gaps between marriage and childbirth were more predictive of TNBC. Conclusions: Our findings underscore the value of machine learning coupled with SHAP-based explainability in identifying context-specific risk factors for breast cancer subtypes in resource-limited settings. This approach enhances transparency and supports the development of targeted public health interventions to reduce breast cancer disparities in Bangladesh.

## 1. Introduction

Breast cancer is an escalating public health concern globally, but its incidence in Bangladesh indicates unique challenges associated with the country’s socio-economic, healthcare, and cultural landscape [1,2,3]. This increase in breast cancer cases, while aligned with global trends, poses significant burdens on its resource-limited healthcare infrastructure. Notably, breast cancer cases in Bangladesh are frequently present in the advanced stages [4]. Limited screening programs and delayed healthcare-seeking behavior contribute to poorer survival rates and higher mortality compared to more developed countries, further compounding national healthcare challenges [5].

While reproductive factors—such as age at menarche, age at first childbirth, parity, and contraceptive use—are well-established contributors to breast cancer risk, much of the research linking these factors to specific breast cancer subtypes has been conducted in high-income countries [6,7]. These studies often fail to account for the distinct reproductive practices and behaviors observed in low- and middle-income settings, such as early marriage, early motherhood, and limited contraceptive use. In Bangladesh, these unique reproductive patterns are shaped by cultural norms and economic factors that potentially influence the etiology of breast cancer.

Specifically, the distribution of hormone-receptor-positive (HR+) and triple-negative breast cancer (TNBC) subtypes differs between populations [8]. HR+ breast cancers, which are driven by hormonal factors and often have better prognoses, tend to be more common in high-income settings where delayed childbearing and widespread contraceptive use are prevalent [9,10,11,12]. In contrast, TNBC, characterized by its aggressive nature, lack of targeted hormonal therapies, and poor survival outcomes, is more frequently observed in younger women and populations with distinct reproductive behaviors, such as those found in Bangladesh [13,14,15,16]. However, the interplay between these reproductive factors and breast cancer subtype prevalence remains underexplored in Bangladesh.

This gap in the literature highlights the critical need to examine the contributions of reproductive and demographic factors to breast cancer risk in Bangladesh, with a focus on HR+ and TNBC subtypes. Despite their distinct clinical implications, little is known about the correlation between these subtypes and reproductive history within low-resource settings. Such insights could significantly advance our understanding of breast cancer etiology in Bangladesh, enabling more tailored prevention strategies and subtype-specific interventions.

This study addresses the critical need for localized data by investigating the relationships among reproductive factors, demographic variables, and breast cancer subtypes among Bangladeshi women. Utilizing advanced machine learning (ML) approaches—including XGBoost, Random Forest, Support Vector Machine, Logistic Regression with Lasso Regularization, and standard Logistic Regression—paired with Shapley Additive exPlanation (SHAP)-based model explainability techniques, this research aims to uncover key predictors for HR+ and TNBC [17]. SHAP leverages game theory to quantify feature importance by first training a classification model on all features and then computing SHAP values for each feature across individual predictions [18,19]. While these values are often aggregated to rank features globally, we retained the instance-level SHAP values to enable a more granular understanding of how specific features influenced the model’s decision making at the patient level.

Thus, by contextualizing these findings within the unique reproductive and socio-demographic landscape of Bangladesh, this study provides a nuanced understanding of the factors influencing breast cancer risk as evidenced by a tertiary cancer hospital. To the best of our knowledge, this is the first investigation of its kind targeting Bangladeshi women. The results hold significant potential to fill existing knowledge gaps, guide evidence-based public health policies, and improve clinical strategies, thereby contributing to global efforts to reduce disparities in breast cancer prevention and outcome.

## 2. Methods

### 2.1. Study Design and Setting

This case-control study was conducted at the National Institute of Cancer Research and Hospital (NICRH), a public tertiary care hospital in Dhaka, Bangladesh. NICRH is the only government-designated tertiary referral center for cancer care in the country and serves as the principal institution for oncology services nationwide. As a centralized public facility, NICRH receives cancer patients from all administrative divisions of Bangladesh, thus providing a nationally representative patient population. The hospital offers multidisciplinary cancer care, including diagnosis, treatment, and palliative services. Data for this study were collected between January 2021 and December 2021.

### 2.2. Study Population

The study included 929 Bangladeshi women aged 18 to 75 years, comprising 486 cases and 443 age-matched controls. Patients with histopathologically confirmed breast cancer were treated at the NICRH during the study period. Controls were cancer-free women selected from among hospital visitors accompanying patients in other departments. Controls were matched with cases by age within a ±5-year range and district of residence to account for regional disparities in healthcare access.

### 2.3. Inclusion and Exclusion Criteria

- Cases: Women with confirmed histopathological diagnoses of breast cancer. The exclusion criteria included a history of other malignancies or incomplete medical records.
- Controls: Women without a history of cancer. The exclusion criteria were malignancies or reproductive health conditions that could confound the analysis.

### 2.4. Data Collection

Data were collected through structured interviews and medical records. A team of six trained physicians conducted face-to-face interviews following standardized protocols to minimize bias. For participants with no literacy, the consent form was read aloud in the presence of a family member. To ensure high data quality, the data collectors received comprehensive training on the study protocols and collection techniques. Instruments were pretested on a similar population to address potential issues prior to formal data collection. Field supervisors conducted periodic quality checks for completeness and accuracy, resolving discrepancies or missing data through follow-up with the collection team.

## 3. Variables Assessed

### 3.1. Primary Outcome Variable

The primary outcome variable was the breast cancer diagnosis, which was confirmed by histopathology. Patients were classified into three groups, “No Cancer,” “ TNBC,” and “HR+”, to reflect the two major breast cancer subtypes examined in this study.

### 3.2. Independent Variables

- Socio-Demographic Factors: Age, education level (0–5 years, 6–12 years, 13–20 years), residential area (rural, urban, metropolitan), and employment status (unemployed, employed).
- Reproductive Factors: Age at menarche, age at first marriage, age at first childbirth, gaps between menarche and first childbirth, and first marriage and first childbirth, parity (categorized as 0, 1, or >1 child), type of delivery (vaginal, cesarean, or both), menstrual regularity (regular or irregular), menopausal status (premenopausal or postmenopausal), and abortion history (categorized as no abortion, 1, or ≥2 abortions).
- Anthropometric Measures: Body mass index (BMI) was calculated using height and weight measurements and categorized as underweight (<18.5 kg/m^2^), normal (18.5–24.9 kg/m^2^), and obese (≥25 kg/m^2^) based on WHO guidelines.

### 3.3. Experimental Design

A machine learning-based predictive analysis was conducted to identify the features most associated with breast cancer subtypes, including TNBC and HR+ cases. The experimental design included comprehensive preprocessing, the application of diverse machine learning models, and rigorous evaluation using stratified cross-validation and performance comparison metrics. Shapley values were employed for model explainability to highlight the key predictors of breast cancer subtypes in Bangladeshi women.

### 3.4. Data Preprocessing and Model Training

Numeric variables, including age, age at first marriage, age at first childbirth, age at menarche, and gaps between menarche and first childbirth and first marriage and first childbirth were normalized using min–max scaling to standardize their range. Additionally, socio-demographic and reproductive variables, such as residential area, employment status, parity, delivery type, menstrual status, and nutrition, were one-hot-encoded to facilitate machine learning analysis. The target variable (type) was categorized into three levels: “No Cancer,” “HR+,” and “TNBC.”

For model training and evaluation, the dataset was divided into 80% training and 20% testing subsets using stratified sampling to maintain the class balance across the subcategories. Stratified five-fold cross-validation was employed during training to ensure robust evaluation and mitigate overfitting, while preserving class proportions within each fold. This approach ensured that the models are trained using representative and balanced data.

Five machine learning models were trained and evaluated to ensure a comprehensive analysis. Logistic Regression was used as a baseline model, whereas Logistic Regression with Lasso Regularization added feature selection by penalizing less important predictors. Support Vector Machines (SVMs) with a radial kernel handling nonlinear relationships in the data, Random Forest that built an ensemble of decision trees for robust predictions, and XGBoost that leveraged gradient boosting were employed. The performance of each model was evaluated using metrics derived from confusion matrices, including sensitivity, specificity, precision, recall, F1-score, and balanced accuracy. Additionally, the multiclass Area Under the Receiver Operating Characteristic (ROC) Curve (AUC) was calculated to assess the predictive power of the model for each breast cancer subtype.

### 3.5. Explainability of ML Models

To ensure robust and interpretable model predictions, we employed Shapley Additive exPlanation (SHAP) values to analyze feature importance for each breast cancer subtype. SHAP, originating from cooperative game theory, quantifies the contribution of individual features to model output by computing per-observation values. The method first trains a classification model using all available features and then calculates SHAP values for each feature across all instances, enabling the evaluation of both global and local (individual-level) feature impacts. SHAP is model-agnostic and supports consistent additive attribution, making it a powerful tool for post hoc explainability in machine learning applications [18,19].

In our study, SHAP values were computed using the best-performing model for each breast cancer subtype to ensure that feature importance interpretations reflect the most accurate and subtype-specific predictive behavior. SHAP values were computed for every observation–feature pair using the ‘fastshap’ package in R. While SHAP values are often aggregated across all samples to derive global feature importance, we retained and systematically analyzed the individual-level values to investigate how socio-demographic and reproductive factors influenced model predictions at the patient level.

Three complementary approaches were used to interpret and visualize these values:

a. Global Feature Importance with Confidence Intervals: We averaged observation-level SHAP values for each feature to obtain global importance rankings for HR+ and TNBC. These average values, along with 95% confidence intervals, were plotted to show the overall direction and magnitude of each feature’s contribution. A positive average indicates a driving effect on the prediction, while a negative value indicates a suppressive effect. To statistically assess which features contributed significantly to subtype predictions, we applied the Wilcoxon signed-rank test. Features with mean SHAP values significantly different from zero (*p* < 0.05) were highlighted as key predictors.
b. Comparative Analysis of Feature Importance: To assess whether feature importance differed between subtypes, we performed pairwise comparisons of SHAP values for HR+ and TNBC cases using the Wilcoxon signed-rank test. Features were categorized as more predictive for TNBC (μ_TN > μ_HR+), more predictive for HR+ (μ_TN < μ_HR+), or equally predictive (μ_TN = μ_HR+). This comparison enabled the identification of subtype-specific versus shared predictors.
c. Visualization of Trends in Numeric Features: To explore how the predictive impact of numeric features varied across their range, we plotted individual-level SHAP values against the actual feature values. Separate scatterplots were created for each subtype, with trend lines depicting smoothed averages. These visualizations revealed nonlinear patterns—for example, the decreasing importance of age at menarche with increasing age for HR+ cases—highlighting how SHAP can reveal meaningful trends that conventional coefficients or feature weights may miss.

The use of individual-level SHAP values allowed us to go beyond average effects and understand the nuanced patient-specific ways in which features shaped model predictions. This framework provided a transparent, interpretable, and statistically rigorous foundation for evaluating the socio-demographic and reproductive determinants of breast cancer subtypes in our population.

### 3.6. Ethical Considerations

The study protocol was approved by the National Institute for Cancer Research and Hospital Institutional Review Board (Ref: NICRH/Ethics/2021/89). Written informed consent was obtained from all the participants. Data confidentiality and anonymity were strictly maintained, and all data were stored securely in a password-protected database.

## 4. Results

### 4.1. Study Population Characteristics

The cohort consisted of 443 women without any breast cancer diagnosis, 246 HR+ cases, and 240 TNBC cases (Table 1). The majority of HR+ and TNBC patients resided in rural areas (76.4% and 60.8%, respectively), whereas a larger proportion of cancer-free individuals lived in metropolitan regions (53.7%). Employment status, education level, and reproductive factors showed significant variation across subtypes, with higher employment among the cancer-free group (20.8%) compared to the HR+ (2.0%) and TNBC (6.7%) groups.

Women with HR+ and TNBC subtypes had lower educational attainment (80.5% and 79.2% had ≤5 years of education, respectively) compared to the cancer-free group (20.1%). Reproductive factors also varied significantly, with patients with TNBC having the highest rate of multiple abortions (9.2% with ≥2 abortions) and a higher proportion of postmenopausal women (59.2%). Menstrual irregularities were more common among HR+ (30.5%) and TNBC (32.9%) patients than in cancer-free women (16.7%).

Regarding age-related factors, TNBC cases had the highest mean age (45.0 ± 10.2 years), followed by HR+ (43.0 ± 10.4) and cancer-free individuals (41.0 ± 11.4) (*p* < 0.001). Similarly, the age at menarche was significantly higher in TNBC patients (13.03 years) than in HR+ (12.05 years) and cancer-free individuals (12.64 years) (*p* < 0.001). The gaps between reproductive milestones, such as menarche to first childbirth and first marriage to first childbirth, were longer in patients with TNBC (*p* = 0.048 and *p* = 0.082, respectively), indicating distinct reproductive patterns.

### 4.2. Model Comparison

**Figure 1:**
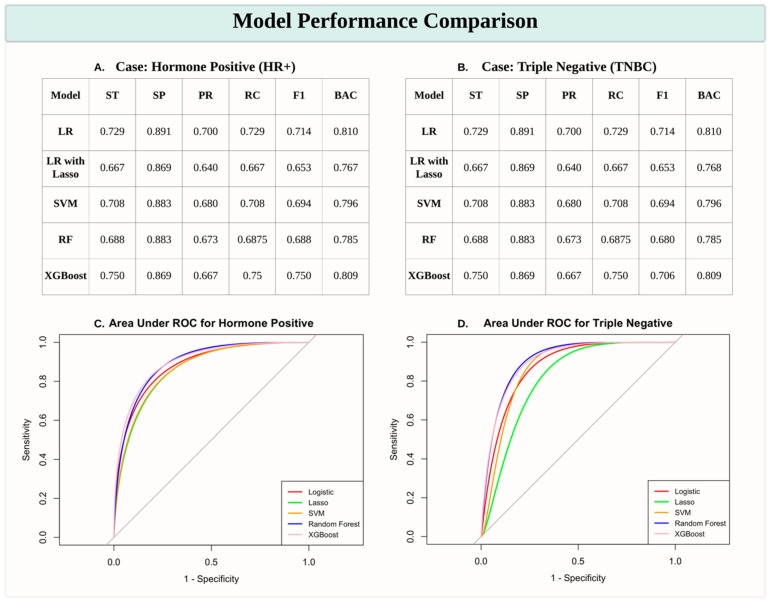
Model performance comparison for predicting breast cancer subtypes. (**A**,**B**) Tables presenting performance metrics for predicting HR+ and TNBC subtypes across various machine learning models, including Logistic Regression (LR), Logistic Regression with Lasso Regularization (LR with Lasso), Support Vector Machine (SVM), Random Forest (RF), and XGBoost. Metrics include sensitivity (ST), specificity (SP), precision (PR), recall (RC), F1 score (F1), and balanced accuracy (BAC). (**C**,**D**) Receiver Operating Characteristic (ROC) curves with Area Under the Curve (AUC) scores for HR+ (**C**) and TNBC (**D**) predictions, illustrating model performance in distinguishing breast cancer subtypes. XGBoost consistently demonstrates robust performance, particularly for HR+ cases.

The performance of various machine learning models in predicting the HR+ and TNBC subtypes was assessed using sensitivity (ST), specificity (SP), precision (PR), recall (RC), F1 score (F1), and balanced accuracy (BAC). Among the models, XGBoost consistently demonstrated superior performance, achieving the highest sensitivity (0.750 for both HR+ and TNBC cases as shown in Figure 1A,B) and F1 scores (0.750 and 0.706 for HR+ and TNBC, respectively), indicating its robustness in handling data complexity. Logistic Regression (LR) also performed well, with comparable balanced accuracy (0.810 for both subtypes), while Logistic Regression with Lasso Regularization (LR with Lasso) showed relatively lower scores across most metrics. The Support Vector Machine (SVM) and Random Forest (RF) models achieved moderate performance, with balanced accuracy ranging from 0.785 to 0.796. ROC curves further validated the effectiveness of these models, with XGBoost exhibiting the highest Area Under the Curve (AUC) values, particularly for HR+ predictions (Figure 1C,D). These results highlight that XGBoost is the most effective model for distinguishing breast cancer subtypes in this study.

### 4.3. Feature Importance and Comparative Analysis

**Figure 2:**
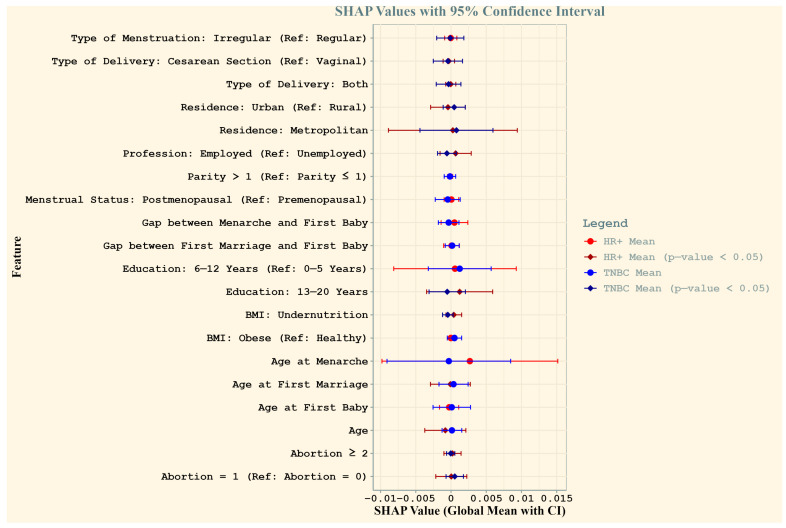
Comparison of SHAP values for HR+ and TNBC cases among Bangladeshi women. The plot illustrates the global mean SHAP values (dots) with 95% confidence intervals (horizontal lines) for each feature, providing insights into their contribution to the prediction of breast cancer subtypes. Red represents HR+ cases and blue represents TNBC cases, with bold vertical lines denoting significance (*p*-value < 0.05) based on Wilcoxon signed-rank tests. Features with significant SHAP values are highlighted with distinct diamond markers. Feature names reflect reproductive and socio-demographic factors critical to breast cancer risk in the study population.

Figure 2 compares the feature importance for HR+ and TNBC cases among Bangladeshi women. The global mean SHAP values, with 95% confidence intervals, highlight features that influence subtype predictions. Significant predictors (*p*-values < 0.05) for both subtypes included the type of delivery (both vaginal and cesarean), residence, employment status, education level (13–20 years), BMI (undernutrition), and abortion history. Age at first marriage and age emerged as uniquely significant predictors of HR+ cases, reflecting subtype-specific demographic and reproductive patterns.

**Figure 3:**
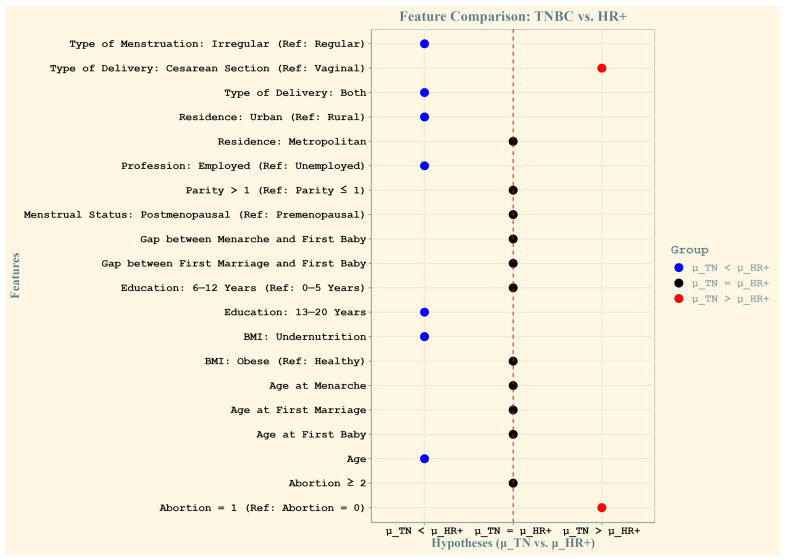
Relative contributions of features to the prediction of triple-negative (TN) and HR+ breast cancer cases among Bangladeshi women. The *x*-axis represents the three tested hypotheses using Wilcoxon paired comparisons: μ_TN > μ_HR+ (greater importance for TN cases), μ_TN = μ_HR+ (no difference in importance), and μ_TN < μ_HR+ (greater importance for HR+ cases). The *y*-axis displays the features, with their labels positioned relative to their contribution. Blue dots indicate features that are more important for predicting triple-negative cases, while red dots represent features that are more important for hormone-receptor-positive cases. The central black dots are features for which the average importance is not significantly different between TN and HR+ cases, suggesting equal relevance for both subtypes.

Figure 3 illustrates the relative contributions of socio-demographic and reproductive features in predicting TNBC and HR+ cases among Bangladeshi women. The *x*-axis represents the three tested hypotheses using Wilcoxon paired comparisons: μ_TN > μ_HR+ (greater importance for TNBC), μ_TN = μ_HR+ (equal importance), and μ_TN < μ_HR+ (greater importance for HR+). The *y*-axis lists the features, and their positions indicate their relative contribution. Blue dots represent features with greater importance for TNBC prediction, whereas red dots indicate features more relevant for HR+ cases. Black dots signify features with no significant difference in contribution between subtypes.

The key findings include a greater mean importance for HR+ cases for features such as irregular type of menstruation, both vaginal and cesarean delivery types, urban residence, employment status, higher education levels (13–20 years), undernutrition (low BMI), and age. Conversely, cesarean delivery and a history of two or more abortions were significantly more important predictors of TNBC. Features represented by black dots, such as other types of menstruation and reproductive factors, showed equal relevance across both subtypes. This analysis highlights the distinct and shared predictive patterns for the two subtypes of breast cancer.

**Figure 4:**
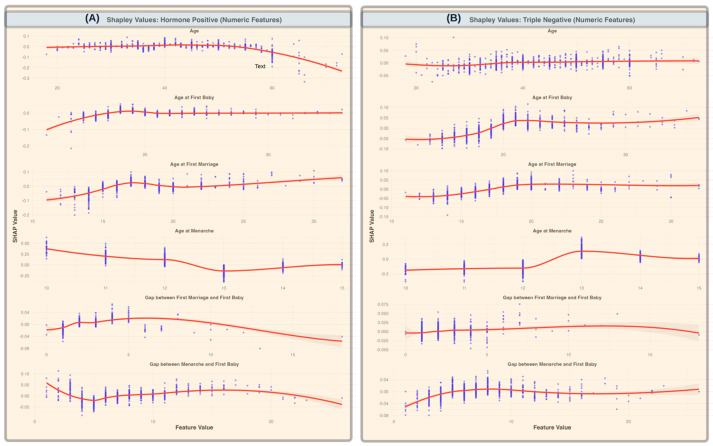
SHAP values for numeric features across breast cancer subtypes (**A**). SHAP values for HR+ cases, illustrating the impact of numeric features, such as age, age at first baby, age at first marriage, age at menarche, gap between first marriage and first baby, and gap between menarche and first baby, on model predictions. (**B**) SHAP values for TNBC cases, highlighting the same numeric features. The scatter points represent individual SHAP values for each data instance, while the red trend lines represent the smoothed averages (with standard deviations shaded) across feature values, showing the overall contribution trends of each feature. A decreasing trend in importance for age at menarche in HR+ cases is observed, indicating reduced predictive importance with increasing age. In contrast, gaps between first marriage and first baby show increasing predictive relevance for TNBC cases as the gap widens.

Figure 4 highlights key numerical features influencing breast cancer subtype predictions by showing how SHAP values vary with change in the feature value. For HR+ cases (Panel A), age-related features, such as age at menarche and age at first birth, showed a decreasing trend in predictive importance as values increase, suggesting that younger ages at these reproductive milestones contribute more to model predictions. The gap between first marriage and first birth exhibits a relatively stable trend, with a slight increase at wider gaps. In contrast, for TNBC cases (Panel B), an opposite trend was observed for the gap between first marriage and first baby, where an increasing gap was associated with stronger predictive importance. Additionally, age-related features, such as age at first birth and age at menarche, exhibit varying levels of importance across different values.

## 5. Discussion

Our study provides crucial insights into the predictors of HR+ and TNBC subtypes among Bangladeshi women, highlighting distinct risk factors that differ from those observed in other high-income countries. The application of machine learning models enabled a robust analysis of these risk factors and provided valuable insights into their contribution in HR+ and TNBC classification. Among the models tested, XGBoost demonstrated the highest predictive accuracy, supporting its utility in clinical decision making for breast cancer diagnosis and subtype classification. The integration of the SHAP values allowed for explainability in our models, identifying key predictors for each breast cancer subtype. Our findings highlight the critical role of socioeconomic and reproductive factors in shaping breast cancer subtypes among Bangladeshi women, revealing both alignments and divergences with global trends observed in developed nations. While the established literature in high-income countries emphasizes delayed childbearing, prolonged hormonal exposure, and obesity as primary risk factors for breast cancer [20], our study suggests a more nuanced interplay of economic, reproductive, and demographic determinants in Bangladesh.

### 5.1. Socioeconomic Disparities and Breast Cancer Risk

One of the key findings of this study is the strong association between residence in a metropolitan area and an increased risk for both HR+ and TNBC. This aligns with global patterns where urbanization is linked to increased breast cancer incidence due to lifestyle factors such as diet, pollution, and delayed reproduction [21]. However, a unique observation is that residing in an urban (non-metropolitan) area is predictive of TNBC but appears to be protective for HR+. This suggests that the urban–rural divide in Bangladesh may reflect distinct environmental and healthcare access disparities that differ from those of developed nations, where urbanization is generally associated with an overall increase in breast cancer risk.

Employment status also emerged as a significant factor, with employment predicting a higher risk of HR+ while being protective for TNBC. This is in contrast to findings from developed nations, where employment is often correlated with better healthcare access and preventative care, potentially reducing breast cancer risk [22]. In Bangladesh, this trend may reflect occupational stress, exposure to environmental toxins, or differences in access to reproductive healthcare among working women.

Education level also demonstrated an intriguing trend: higher education (13–20 years) was predictive of HR+ but protective for TNBC. This deviates from the patterns seen in high-income countries, where higher education typically correlates with lower TNBC breast cancer risk due to better health awareness and access to medical care [23]. In Bangladesh, higher education may be linked to delayed childbirth and changes in reproductive behavior, thereby increasing HR+ risk while reducing exposure to risk factors associated with TNBC.

### 5.2. Reproductive Health and Breast Cancer Subtypes

Reproductive factors play a crucial role in shaping breast cancer risk, with BMI undernutrition being significantly predictive of HR+ but protective for TNBC. This contrasts with developed nations, where obesity is a well-established risk factor for HR+, whereas undernutrition is rarely considered a major determinant [24]. The protective effect of undernutrition on TNBC suggests that metabolic and nutritional deficiencies may influence the aggressive nature of breast cancer subtypes differently in South Asian populations.

Cesarean section as a mode of delivery was found to be protective against TNBC, whereas abortion (≥1) was predictive of TNBC risk. This contradicts global trends, in which reproductive surgeries and terminations have not shown consistent subtype-specific effects [25]. The association between cesarean delivery and reduced TNBC risk may reflect underlying health-seeking behaviors or unmeasured confounders such as gestational health complications that influence long-term cancer risk.

Age-related reproductive factors showed distinct trends. Age at menarche and age at first childbirth showed a decreasing trend in predictive importance with increasing values, suggesting that a younger reproductive age is more strongly associated with HR+ risk. This is in line with studies in developed nations that link early menarche and early first childbirth to prolonged hormonal exposure, thereby increasing the HR+ incidence [26,27,28,29]. However, the gap between first marriage and first childbirth demonstrated the opposite trend; an increasing gap was strongly predictive of TNBC. This pattern has not been widely documented in developed nations and may reflect cultural and societal pressures in Bangladesh, where extended gaps between marriage and childbirth may be influenced by economic instability, infertility, or prolonged exposure to social stressors. These findings contribute to the growing body of literature on breast cancer epidemiology in low- and middle-income countries (LMICs) and underscore the urgent need for targeted prevention and treatment strategies tailored to the Bangladeshi population.

## 6. Challenges and Policy Recommendations

One of the major challenges in conducting this study was the lack of a centralized electronic health record (EHR) system in Bangladesh. The absence of standardized medical records makes it difficult to track patient histories, perform longitudinal analyses, and ensure accurate data. Instead, data collection relied on patient interviews and self-reported reproductive histories, which may have introduced a recall bias. Developing a national cancer registry can help mitigate these issues by systematically documenting cancer cases, treatment outcomes, and risk factors.

Additionally, genetic testing and molecular profiling are not widely available in Bangladesh, which limits our ability to incorporate genetic risk factors into the analysis. In developed countries, genetic predispositions, such as BRCA mutations, are key determinants of breast cancer risk, particularly in TNBC cases. Expanding access to genetic testing could provide more comprehensive insights into hereditary cancer risk in Bangladesh and inform personalized treatment approaches.

Our findings underscore the need for tailored breast cancer prevention and treatment strategies in Bangladesh. Given the observed socioeconomic and reproductive disparities, public health interventions should prioritize education on reproductive health, nutrition, and cancer screening programs, particularly for women in metropolitan areas. The association between employment and HR+ risk suggests the need for workplace health policies that promote cancer awareness and facilitate early detection programs.

Furthermore, the distinct reproductive patterns in Bangladesh necessitate localized treatment guidelines that account for factors such as early menarche, a high prevalence of cesarean deliveries, and extended gaps between marriage and first childbirth. Unlike treatment approaches in developed nations, which focus on risk mitigation through hormonal therapies and lifestyle modifications, Bangladeshi women may benefit from interventions that address nutritional deficiencies, metabolic health, and culturally specific reproductive practices.

## 7. Limitations and Future Research Directions

While this study offers novel insights into the socio-demographic and reproductive determinants of breast cancer subtypes among Bangladeshi women, several limitations should be acknowledged. First, the data were collected from a single institution, which, despite being the only government-designated tertiary referral center for cancer care in Bangladesh and drawing patients from across the country, may still limit generalizability to other regional or international populations. We recommend future multi-center studies to assess the consistency of these findings across different healthcare settings.

Second, the retrospective case-control design relies heavily on self-reported socio-reproductive information, which may be affected by recall bias and cultural stigma—particularly for sensitive topics such as abortion or contraception use—leading to possible misclassification or underreporting. Furthermore, the cross-sectional nature of data collection precludes causal inference; observed associations cannot confirm temporal relationships.

Third, our analysis focused solely on non-biological predictors, without incorporating genetic, molecular, or hormonal markers, which are known to influence cancer prevalence in general [30,31,32,33]. Future research should seek to combine socio-demographic and behavioral data with biological features to enhance risk prediction and subtype classification. Our work provides early insights into the predictive relevance of socio-reproductive factors and may inform future integrative research combining these variables with biological markers, particularly as more comprehensive resources like EHR-linked biorepositories become available in low-resource settings such as Bangladesh.

Fourth, although machine learning models such as XGBoost demonstrated strong predictive performance in our analysis, the evaluation was limited to internal validation using stratified five-fold cross-validation. To ensure the generalizability and robustness of these models across diverse clinical contexts, future research should prioritize external validation using independent datasets from different populations and healthcare settings. This step is critical before such models can be reliably translated into clinical practice for subtype-specific breast cancer risk assessment.

Despite these limitations, our study demonstrates the promise of explainable AI techniques, such as SHAP values, in explaining individualized and subtype-specific risk factors. Moving forward, we recommend exploring the real-world clinical integration of ML models in LMIC contexts. These efforts should prioritize interpretability, local adaptability, and accessibility to ensure the equitable benefits of AI-driven cancer risk assessment tools in resource-limited healthcare environments.

## 8. Conclusions

This study provides novel insights into the distinct socio-demographic and reproductive predictors of HR+ and TNBC subtypes in Bangladeshi women. Our findings highlight the importance of tailored public health interventions, improved healthcare accessibility, and the potential of machine learning models for breast cancer risk prediction. Addressing the disparities identified in this study through policy changes, increased awareness, and enhanced screening efforts could lead to better breast cancer outcomes in Bangladesh. Further research is needed to explore the underlying biological mechanisms driving these associations and to develop interventions that bridge the gap between global breast cancer prevention guidelines and local epidemiological realities.

## Figures and Tables

**Table 1:** Distribution of socio-demographic and reproductive characteristics across breast cancer subtypes (No Cancer, HR+, and TNBC) in the study population. The categorical variables are presented as counts and percentages *n (%)*, while continuous variables are shown as mean (standard deviation). *p*-values correspond to chi-square tests for categorical variables and ANOVA for continuous variables, assessing differences across the three subtypes.

	Breast Cancer Subtypes			
Features	No Cancer	HR+	TNBC	*p*-Value
Breast Cancer Subtype	443 (100%)	246 (100%)	240 (100%)	
**Residential Area (*n* (%))**				
Rural	98 (22.12%)	188 (76.42%)	146 (60.83%)	<0.05
Urban	107 (24.15%)	48 (19.51%)	80 (33.33%)	<0.05
Metropolitan	238 (53.72%)	10 (4.07%)	14 (5.83%)	<0.05
**Employment Status**				
Unemployed	351 (79.23%)	241 (97.97%)	224 (93.33%)	<0.05
Employed	92 (20.77%)	5 (2.03%)	16 (6.67%)	<0.05
**Education Level**				
0–5 Years	89 (20.09%)	198 (80.49%)	190 (79.17%)	<0.05
6–12 Years	243 (54.85%)	37 (15.04%)	37 (15.42%)	<0.05
13–20 Years	111 (25.06%)	11 (4.47%)	13 (5.42%)	<0.05
**Parity**				
<=1	254 (57.34%)	131 (53.25%)	119 (49.58%)	0.142
>1	189 (42.66%)	115 (46.75%)	121 (50.42%)	0.142
**Type of Delivery**				
Normal Vaginal Delivery (NVD)	258 (58.24%)	188 (76.42%)	163 (67.92%)	<0.05
Cesarean Section (CS)	144 (32.51%)	31 (12.6%)	25 (10.42%)	<0.05
Both Type (CS + NVD)	41 (9.26%)	27 (10.98%)	52 (21.67%)	<0.05
**Type of Menstruation**				
Regular	369 (83.3%)	171 (69.51%)	161 (67.08%)	<0.05
Irregular	74 (16.7%)	75 (30.49%)	79 (32.92%)	<0.05
**Menstrual Status**				
Premenopausal	276 (62.3%)	130 (52.85%)	98 (40.83%)	<0.05
Postmenopausal	167 (37.7%)	116 (47.15%)	142 (59.17%)	<0.05
**Abortion**				
No	339 (76.52%)	148 (60.16%)	177 (73.75%)	<0.05
Yes = 1	89 (20.09%)	84 (34.15%)	41 (17.08%)	<0.05
Yes > = 2	15 (3.39%)	14 (5.69%)	22 (9.17%)	<0.05
**Body Mass Index (BMI)**				
Healthy	138 (31.15%)	107 (43.5%)	98 (40.83%)	<0.05
Obese	298 (67.27%)	127 (51.63%)	127 (52.92%)	<0.05
Undernutrition	7 (1.58%)	12 (4.88%)	15 (6.25%)	<0.05
**Age (Mean (SD))**	41.05 (11.4)	43.00 (10.4)	45.04 (10.2)	<0.05
**Age at First Marriage**	17.73 (3.54)	17.46 (3.00)	18.55 (3.19)	<0.05
**Age at First Baby**	19.80 (3.82)	19.57 (3.29)	20.91 (3.65)	<0.05
**Age at Menarche**	12.64 (1.09)	12.05 (1.21)	13.03 (0.37)	<0.05
**Gap Between Menarche and First Baby**	7.17 (3.84)	7.52 (3.26)	7.88 (3.59)	0.048
**Gap Between First Marriage and First Baby**	2.07 (1.71)	2.11 (1.42)	2.36(1.78)	0.082

## Data Availability

The data are not publicly available due to privacy or ethical restrictions.

## Code Availability

https://github.com/hikf3/ExplainableML-BreastCancerRisk-Bangladeshi-Women. (accessed on 13 June 2025).
